# The Role of Autophagy in Varicella Zoster Virus Infection

**DOI:** 10.3390/v13061053

**Published:** 2021-06-02

**Authors:** Johanna Heinz, Peter G. E. Kennedy, Trine H. Mogensen

**Affiliations:** 1 Department of Infectious Diseases, Aarhus University Hospital, 8000 Aarhus, Denmark; Joheinz@biomed.au.dk (J.H.); trine.mogensen@biomed.au.dk (T.H.M.); 2 Department of Biomedicine, Aarhus University, 8000 Aarhus, Denmark; 3 Institute of Infection, Immunity and Inflammation, University of Glasgow, Glasgow G61 1QH, UK

**Keywords:** varicella zoster virus, autophagy, latency, pathogen, phagosome, endoplasmic reticulum, central nervous system

## Abstract

Autophagy is an evolutionary conserved cellular process serving to degrade cytosolic organelles or foreign material to maintain cellular homeostasis. Autophagy has also emerged as an important process involved in complex interactions with viral pathogens during infection. It has become apparent that autophagy may have either proviral or antiviral roles, depending on the cellular context and the specific virus. While evidence supports an antiviral role of autophagy during certain herpesvirus infections, numerous examples illustrate how herpesviruses may also evade autophagy pathways or even utilize this process to their own advantage. Here, we review the literature on varicella zoster virus (VZV) and autophagy and describe the mechanisms by which VZV may stimulate autophagy pathways and utilize these to promote cell survival or to support viral egress from cells. We also discuss recent evidence supporting an overall antiviral role of autophagy, particularly in relation to viral infection in neurons. Collectively, these studies suggest complex and sometimes opposing effects of autophagy in the context of VZV infection. Much remains to be understood concerning these virus–host interactions and the impact of autophagy on infections caused by VZV.

## 1. Varicella Zoster Virus Infection

Varicella Zoster virus (VZV) is a human herpesvirus that causes varicella (chicken pox) as a primary infection, and following a variable latent period of up to several years, it may reactivate to cause herpes zoster (shingles), which is a painful vesicular rash occurring within the distribution of a specific sensory dermatome [1,2]. During the period of viral latency, VZV is present in neurons in the peripheral ganglia along the entire neuroaxis, especially the dorsal root ganglia and trigeminal ganglia, although the peripheral autonomic ganglia may also harbor latent VZV [1,3,4]. When VZV reactivates, this may occur spontaneously or after different triggering events, such as infection, trauma, increasing host age, malignancy, X-ray irradiation or immunosuppression, the latter resulting from either disease or drug therapy [1,5]. Although the most serious complication of herpes zoster is post-herpetic neuralgia, which is a very painful and persistent condition that is frequently unresponsive to treatment, it has been increasingly recognized that the clinical spectrum of VZV reactivation is more extensive than previously thought since it may produce a wide variety of acute, subacute and chronic neurological conditions, notably, VZV encephalitis and vasculitis [1,6].

Like other herpesviruses, VZV is a double-stranded DNA virus, but it grows only in human cells in which it is highly cell-associated [1]. The VZV genome is thought to contain nearly 125,000 base pairs and 68 open reading frames (ORF)s [1]. However, a recent detailed study has suggested that this may be an underestimate and that the full coding potential of VZV remains unknown [7]. By using a genome-wide transcriptome analysis, it was concluded that VZV may potentially encode more than 68 ORFs. Additionally, the presence of a transcript does not necessarily reflect the ORF coding potential. The mechanisms of latency are still not fully understood, although they are the focus of much current research. During latency, VZV is known to be present in neurons in an episomal configuration [4]. While it is accepted that viral gene expression during latency is restricted, the actual degree of this restriction remains unclear. Originally, it was suggested that VZV *ORFs* *29, 21 62, 63* and *66* were all transcribed in neurons during ganglionic latency [8,9,10], but more recent post-mortem analyses of ganglia that were studied soon after death (6–9 h) reported a transcription of only VZV *ORF 63*, and a newly described spliced VZV transcript (VLT) that was antisense to VZV *ORF 61* [11]. A recent study provided further clarification of the presence of these two transcripts [12], showing that during reactivation from latency, a *VLT-ORF 63* fusion transcript induces broad viral gene expression.

## 2. Autophagy and Its Discovery

Autophagy is an evolutionary, highly conserved degradation pathway in eukaryotic cells, which has been recognized for approximately 60 years [13,14]. In the years following the discovery of this phenomenon, a number of morphological and regulatory studies further elucidated the process (extensively reviewed elsewhere [15]). Of special note is the discovery of specific autophagy related proteins (ATG)s in yeast, many of which are highly conserved in mammals and have human orthologues [16,17]. The autophagy network consists of a variety of pathways, each ultimately leading to the degradation of intracellular content. Currently, three main types of autophagy are recognized: macro-autophagy, micro-autophagy and chaperone-mediated autophagy. Of these, macro-autophagy (hereafter referred to as autophagy) has been the most extensively described, both in the context of viral infections as well as independently of these [18].

**Figure 1:**
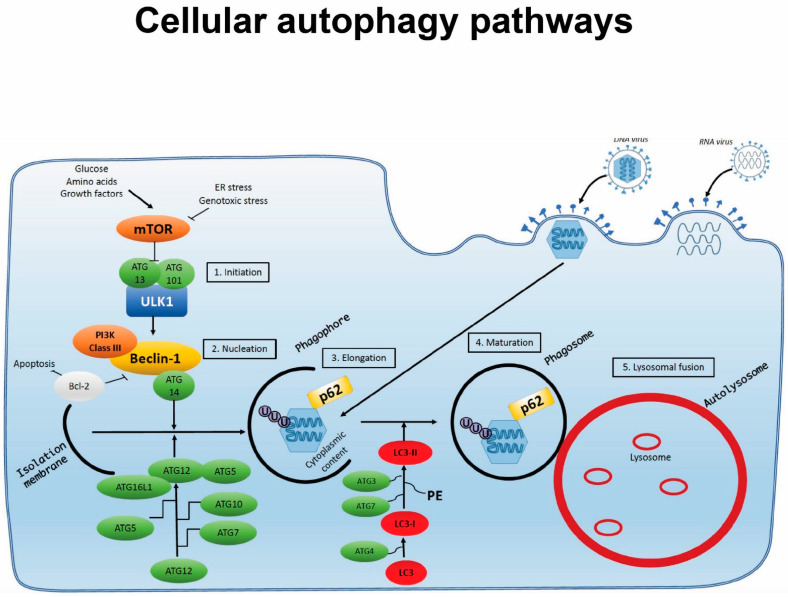
Cellular autophagy pathways. Autophagy is induced by cellular starvation, stress or microbial infection. The process involves the following steps: (1) initiation; (2) nucleation; (3) elongation; (4) maturation; and (5) lysosomal fusion, through which a double-membraned phagosome encapsulating cytosolic material is targeted for degradation after fusion with lysosomes to form an autolysosome. During infection, pathogens may activate autophagy pathways but also have the capacity to interfere with and exploit this process through interaction with specific autophagy proteins or steps in the autophagy process.

Autophagy consists of following steps: (1) initiation, (2) nucleation and assembly, (3) elongation, (4) maturation, and (5) degradation (Figure 1). In the canonical pathway, the process is initiated by formation of the unc-51-like-kinase (ULK) complex following stimulation. It consists of proteins ULK1, ULK2 and ATG13, as well as focal adhesion kinase family-interacting protein (FIP) 200 and ATG101 [19,20]. Together, these inhibit the autophagy inhibitor mechanistic target of rapamycin (mTOR) [21]. The inhibition of mTOR in turn activates the formation of the beclin (BECN) complex, composed of BECN1, phosphatidylinositol 3-kinase catalytic subunit type 3 (PIK3C3), p150 and ATG14L, to initiate the nucleation and assembly of the first pre-autophagic membrane, the phagophore [22,23,24,25,26]. This lipid membrane is believed to originally be derived from the endoplasic reticulum (ER) [27]. The BECN complex further recruits ATG5, ATG12 and ATG16L, which form another complex, as well as microtubule-associated protein light chain (MAP1LC) 3, leading to elongation of the membrane [16,28,29,30,31]. Expansion around potential cargo results in the formation of the double-membraned autophagosome. Autophagosomes may fuse with lysosomes and are subsequently termed autolysosomes, in which the cargo is degraded by acidification and enzymatic degradation [32]. Alternatively, autophagosomes may fuse with endosomes, leading to degradation of the cargo or transport to the plasma membrane [33,34].

## 3. Opposing Roles of Autophagy in Herpesvirus Infections

Although originally understood to be a homeostatic process, a variety of different physiological functions have since been attributed to autophagy. A possible role in inflammation and infection, for example, has been suggested since the 1980s and was eventually confirmed at the beginning of this century via evidence from herpesvirus infection research [35]. Currently, many publications describe the role of autophagy in viral infections, which have been proposed to both influence and be influenced by the autophagy machinery in a complex and sometimes opposing manner [36] (Table 1). Since they are intracellular pathogens, most viruses, including herpesviruses, have been reported to interact with the autophagy pathway, both in vitro and in mouse models [35,37,38]. Of particular interest is the observation that autophagy not only exerts a protective effect against herpes simplex virus 1 (HSV-1) but that it is also actively counteracted by the virus [39,40]. Indeed, the HSV-1 infected cell protein (ICP)34.5 actively inhibits the initiation of autophagy by directly interacting with BECN1, leading to abolished autophagy-mediated virus degradation [38]. Notably, failure to inhibit autophagy due to a mutated protein leads to impaired neurovirulence by the virus when tested in a mouse model, suggesting a prominent role of autophagy as an antiviral mechanism in herpesvirus infection in the central nervous system (CNS) [38]. On the other hand, it has been found that many viruses depend on the cellular autophagy machinery in order to successfully complete their life cycle. Such examples can be found within members of the *Herpesviridae*, namely, pseudorabies virus (PRV) and duck enteritis virus (DEV), which have been demonstrated to utilize autophagy rather than simply avoiding it. Not only does PRV and DEV infection lead to increased autophagic flux, but it has been shown that additional activation of autophagy increases viral replication, whereas inhibition of autophagy hinders their replication [41,42,43].

Given that VZV and HSV-1 belong to the same family of alpha-herpesviruses, it is tempting to speculate that the interaction of virus and autophagy pathways might be very similar for these two viruses. However, the smaller VZV genome does not encode the viral protein ICP34.5, or US11, which is another HSV-1 autophagy-inhibiting protein [53]. Since VZV represents another ubiquitous herpesvirus with a high population prevalence, it may be inferred that the absence of these proteins does not significantly impair the virulence of VZV. Instead, these observations could be construed as suggesting a different mode of interaction between VZV and autophagy as compared to the closely related HSV-1. As indicated in the above-noted observations, there are several examples of both the pro- and anti-viral roles of autophagy in herpesvirus infections.

## 4. Activation of Autophagy Flux during VZV Infection

Upregulation in autophagy after VZV infection in a fibroblast cell line and human melanoma cells starting 48 h after infection has been demonstrated [44]. This upregulation was persistent even when blocking the biosynthesis of viral late γ-gene products, indicating that there is not a single protein that specifically activates the autophagy pathway. Rather, it was suggested that induction of autophagy might represent a cellular response to VZV cellular invasion. This explanation is in line with the previous observation that VZV does not encode for any of the genes known to counteract/subvert activation of the autophagy machinery. Despite the phenotype only becoming apparent at a late stage of infection in cell cultures, autophagy markers can be observed simultaneously with ORF62 in the nucleus. These data have led to the notion that upregulation of autophagy may occur at a relatively early stage in the viral life cycle, both in the fibroblast cell line MRC-5 and in infected keratinocytes from biopsies [45,54]. Several methods for measuring autophagic flux revealed evidence for both early- and late-stage autophagic compartments and (uninterrupted) flux, providing evidence that the process is not interrupted by the virus and, therefore, constitutes complete autophagic flux [48]. These data were obtained in both fibroblasts and melanoma cells (MeWos) and for different strains of VZV—both cell-associated as well as cell-free virus inoculums. Taken together with the observation that autophagosomes appear to be present in biopsies of human zoster vesicles, but not in healthy skin, these findings indicate a more general nature of this phenotype. However, in view of the strong cell-type dependency that has been observed in the context of autophagy, further studies in various different cell or organ cultures are required to better understand this process in greater detail. To date, upregulated autophagy following VZV infection has been described in fibroblasts, mainly MRC-5, but also in primary cells, MeWo cells and keratinocytes [44,52,55]. Furthermore, HeLa cells transfected with VZV glycoproteins presented with an enlarged ER and increased LC3-punctae formation [45]. Importantly, these results, obtained in cell cultures, have been confirmed and extended to human skin biopsies, human skin organ cultures and the severe combined immunodeficiency mouse model transplanted with human skin [45,48,51]. Observations in human skin further add to the biological relevance of the findings, illustrating autophagy to be a factor involved in disease pathogenesis, as confirmed by studies in human skin organ cultures, in which a significant increase in autophagic vesicles upon VZV infection was demonstrated [51]. Notably, there is currently a paucity of information on the role of autophagy in VZV infection in non-skin-derived tissue, e.g., neuronal cells. These observations differ significantly from the HSV-1 data showing viral inhibition and evasion of autophagy, possibly to avoid degradation. An interesting approach to studying the differences and similarities between HSV-1 and VZV was provided by Buckingham et al. in 2018, by using recombinant VZV containing the ICP34.5 gene for infection in fibroblasts. Interestingly, the results did not differ from those of a wildtype VZV; i.e., no inhibition of autophagy occurred. This could suggest a neuron-specific function of ICP34.5 or, alternatively, indicate the existence of interactions between the protein and other genetic elements of HSV-1 that are not present in VZV [51].

In 2018, however, another group published data suggesting that VZV does in fact display an inhibitory effect on autophagic flux [50]. In these experiments, MRC-5 fibroblasts were infected with well-described and commonly used VZV Oka strains, as in previous publications. One difference in the experimental designs of these various studies was the way in which autophagy was induced and inhibited. Graybill et al. (2018) used starvation, which has long been a well-established enhancer of autophagic flux [16,50,56]. Following a combination of starvation and VZV infection, they observed fewer cells displaying autophagic flux than after starvation alone, which might be explained by the virus alleviating the effects of starvation. The proposed explanation is that VZV blocks autophagy, partly opposing the autophagy-inducing effects of starvation on the cells. In direct contrast to the data of the various groups presented above, Graybill et al. (2018) observed decreased viral titers following artificially increased autophagic flux using a starvation or rapamycin treatment and an increased viral titer when the autophagic flux was blocked with bafilomycin [50]. Whereas rapamycin acts as an inhibitor of mTOR, the ATPase inhibitor bafilomycin blocks the autophagosome acidification and fusion with lysosomes [21,57]. The autophagy blocker 3-methyladenine (3MA) is believed to prevent autophagosome formation [58]. However, especially under conditions of longer incubation, it might instead induce autophagy. Moreover, this chemical can exert toxic effects upon cells, indicating that the observed decreased viral titer in the presence of 3-MA treatment could be at least partly explained by poor cell survival [59]. Likewise, trehalose, previously used as an autophagy inducer, might block late-stage autophagy while at the same time inducing the early stages [60]. In addition to its ability to block autophagic flux, bafilomycin has been reported to interrupt VZV envelopment by destroying the Golgi apparatus [57,61]. This could, rather than blocking autophagy, be a possible explanation for the decreased viral titers following bafilomycin treatment, as has been observed by some groups [61]. How this chemical was able to increase viral titers in experiments carried out by a different group remains to be elucidated [50]. Collectively, despite most experiments suggesting an overall proviral role of autophagy, these discrepancies indicate the importance of further research that carefully observes the properties and full range of effects of the particular chemicals and cell types.

## 5. A Prominent Role of the Endoplasmic Reticulum in VZV-Induced Autophagy

Based on several studies, it has been established that activation of autophagic vesicles appears to be intimately linked to ER stress and the unfolded protein response (UPR) [62,63,64]. An early study compared VZV-infected cells to cells treated with tunicamycin, a known chemical inducer of ER stress [65]. Both treatments led to the same phenotype of increased autophagy. Furthermore, when using the combination of VZV infection and tunicamycin, the observed phenotype was not stronger than for each condition alone. Therefore, it was concluded that infection might lead to autophagy upregulation via the same pathway as tunicamycin treatment, and that, importantly, VZV alone greatly stresses the ER [45]. ER stress and the UPR are cellular reactions to stress that may, among other cellular reactions, induce autophagy [66]. In addition to the above-mentioned data, it has also been demonstrated that VZV infection leads to upregulation of genes associated with the UPR [47]. When compared to tunicamycin treatment, VZV-infected cells generally upregulate a smaller number of proteins associated with the UPR. In contrast, many gene products related to apoptosis and degradation of the ER are downregulated by VZV [47]. Especially striking is the increase in cyclic AMP-responsive element binding protein H (CREBH), a transcription factor connected to the sensing of ER stress and lipid synthesis. Genes responsible for cholesterol synthesis are also downregulated, possibly leading to a softer ER, which might have a positive impact on the enlargement of this organelle [47]. As shown by upregulated CREBH and Binding immunoglobulin protein (BiP) expression in HeLa cells transfected with gE, it appears to be the glycoproteins that cause the shift in expression [47]. In summary, these results point in the direction of a more specific gene regulation in VZV-infected cells than in tunicamycin-treated cells. This would contribute to enlargement of the ER in order to accommodate enhanced glycoprotein synthesis while at the same time inhibiting degradation and apoptosis in the host cell.

The results described above provide further support for the notion that VZV-induced autophagy may not be induced by one or more specific viral proteins but rather represents a more general cellular stress response to accumulation of viral glycoproteins. In this manner, autophagy represents a cellular defense mechanism that is not interrupted by the virus. This accumulating evidence may indicate an overall proviral role of autophagy in the case of VZV infection. In keeping with this, chemical inhibition of autophagy with 3MA in VZV-infected MRC-5 fibroblasts and MeWo cells has been shown to reduce the viral titer and detectable levels of glycoprotein E [46]. Experiments on fibroblasts showed impaired spreading of VZV through the monolayer when treated with 3MA [58]. In MeWo cells, however, the cytopathic effect in an infected monolayer was markedly reduced, possibly due to limited virus spread and diminished glycoprotein synthesis, which underscores the cell-type-specific nature of autophagy. These results could also be supported by silencing the autophagy protein ATG5, which is another common way to inhibit autophagy [67]. Treatment with the autophagy inducer trehalose has led to wider virus bands being observed in density gradient purification, as well as an increased amount of gE, indicating that artificially increased autophagy results in increased virus replication, whereas autophagy inhibition has the opposite effect [46,68].

## 6. Divergent Roles of Autophagic Vacuoles Leading to Either VZV Egress or Viral Degradation

The late stages of autophagy are characterized by degradation of the cargo present in autophagosomes [32,33,34]. Given the apparent absence of autophagy evasion/inhibition of VZV, the question arises as to how the virus escapes the fate of degradation, instead making its way to the plasma membrane to exit the cell. In 2020, Girsch et al. [52] were able to demonstrate significantly reduced viral titers in cells defective in autophagy, indicating the role of autophagy in promoting exocytosis of VZV. Further co-localization and co-purification studies demonstrated that the virus can indeed be found in autophagic vesicles. However, the same experiments also uncovered co-localization of viral particles with Ras related protein (Rab)11, which has been described as an endosomal marker linking autophagy with the endosomal pathway [49,69]. Notably, electron microscopy (EM) pictures showed viral particles in single-walled LC3 and Rab11 positive vesicles, suggesting an egress pathway using both parts of the autophagic and the endosomal pathway. The absence of VZV in double-walled autophagic vesicles is intriguing and further suggests limited VZV degradation by autophagy. Comparisons of healthy and autophagy-defective cells also indicated the existence of two distinct egress pathways for VZV. Transmission EM analysis showed that in healthy cells, viral capsids seem to accumulate in large vacuoles containing a high number of particles, whereas in the autophagy-defective cells, only small vacuoles, containing a single viral particle each, appear [52]. Importantly, no signs of xenophagy were observed in either the healthy or the deficient cells. The predominant mode in healthy fibroblasts could be connected to the M6P receptor (M6PR), another molecule characteristic of late endosomes. This might suggest a mode of escape from the cell where VZV is present in autophagosomes, which fuse with late endosomes rather than the lysosome, resulting in transport of the virus to the plasma membrane rather than viral degradation. The small vacuoles containing single VZV vesicles observed in the autophagy-defective cells, on the other hand, were negative for M6PR, indicating a second, autophagy-independent mode of cellular egress. While functional, this pathway of viral egress appears to be significantly less efficient than the autophagy-dependent one, highlighting once again the potentially important role of autophagy in the VZV life cycle.

## 7. Conclusions and Overall Perspectives

Taken together, most studies support the role of autophagy in promoting cell survival and in playing an active part in the VZV life cycle, albeit with opposing roles in pro- and antiviral effector pathways. While skin-derived cells represent an important site of viral replication and remain the most extensively studied cell type, knowledge of VZV and autophagy in various cell types remains incomplete and thus warrants further investigation. Of particular interest would be research into VZV-induced autophagy pathways in neuronal cells, with direct relevance for the clinical presentation of VZV CNS infection, neuronal latency and reactivation.

The importance of autophagy in CNS infections is clear from several disease models and further evidenced by human diseases in which autophagy is impaired or abolished. For example, defective autophagy has been shown to enhance neurodegenerative diseases in murine models, indicating a dominant role of autophagy in the CNS [64,70]. Recently, studies on patients with HSV-2-induced recurrent lymphocytic Mollaret meningitis have begun to shed more light on a potentially important role of autophagy in viral infections and disease pathogenesis in humans. Whole-exome sequencing has revealed mutations in the autophagy genes *LC3B2* and *ATG4A* in such patients, which were associated with impaired HSV-2-induced autophagy, increased viral replication and enhanced cell death in patient fibroblasts and in neuronal cells harboring the autophagy variants [71]. Based on these findings, it was suggested that autophagy may play an important role in antiviral defense in the CNS and that autophagy defects may represent an inborn error of immunity predisposing patients to viral meningitis and encephalitis [71]. 

Autophagy may have arisen as an early antiviral defense mechanism; however, many pathogens seem to have evolved strategies to not only evade these mechanisms but also to employ them for their own benefit [36]. Paradoxically, these opposing mechanisms may occur during infection with a given pathogen. In regard to HSV-1, a number of publications suggest evasion strategies as well as potentially protective roles of autophagy during infection [35,38,39,40]. Other members of the Alphaherpesvirinae, namely, PRV and DEV, which closely resemble VZV, seem to have developed strategies to utilize autophagy for their replication, indicated by increased viral titers upon activation of autophagy [41,42,43]. As is the case with PRV and DEV, VZV lacks the specific genes encoding proteins that interfere with autophagy in HSV-1 infection. This, and many of the above-mentioned results, may indicate that under certain circumstances, VZV may be able to exploit autophagy for its own benefit. Indeed, Grose and colleagues observed reduced VZV replication in the presence of pharmacological autophagy inhibitors, together with facilitated VZV glycoprotein biosynthesis and processing when VZV-induced autophagy was occurring [46]. Thus, even though early steps of autophagy may be initially induced, VZV is able to subsequently inhibit later stages of the autophagy pathway in order to avoid degradation. However, the high degree of cell type specificity is a well-established characteristic of autophagy, and several examples of a proviral response of autophagy in one cell type, and of an antiviral role towards the same virus in a different cell type, have been described. Taken together, the emerging picture is that autophagy generally serves a proviral role in VZV infection while also promoting cell survival, and that in some cellular contexts, particularly in neurons, it may exert antiviral roles.

There remain several key questions related to the activation, regulation and pro- and anti-viral roles of autophagy in VZV infection that require further clarification. This underlines the importance of continued research into this complex cellular mechanism, which may be a ‘double-edged sword’ and has opposing roles, depending on the cell type and the viral pathogen. In this context, it will be necessary to combine different methods in order to illuminate the extensive crosstalk between VZV and the autophagy machinery. Further insights into the role of autophagy in VZV infection, latency, reactivation and antiviral responses will be of importance for an enhanced understanding of the pathogenesis of VZV infections and also to improve the therapeutic and prophylactic options for patients with acute or latent VZV infection.

## Figures and Tables

**Table 1:** Pro- and anti-viral roles of autophagy in VZV infection.

Pro- and Anti-Viral Roles of Autophagy	Cellular Protein Involved	Viral Protein Involved	Cell Type	Reference
Autophagy is upregulated upon VZV infection → most likely proviral	LC3 turnover and punctae formation, p62 degradation	? NOT the late γ-gene products	MRC-5 fibroblasts, MeWo cells, Skin biopsies from human zoster vesicles (mostly infected keratinocytes)	[44]
Autophagy in VZV infection is connected to the UPR and ER stress → most likely proviral	Alternatively spliced XBP1, upregulated CHOP	?	MRC-5 fibroblasts	[45]
Autophagy is upregulated and connected to ER stress → most likely proviral	LC3 punctae formation	Viral glycoproteins E, I, H, L	HeLa cells (transfected with viral glycoproteins)	[45]
Autophagy is upregulated upon VZV infection → most likely proviral	LC3 punctae formation	?	Skin biopsies from human varicella and zoster vesicles (mostly infected keratinocytes)	[45]
Inhibition of autophagy reduces viral titer and viral glycoprotein synthesis, enhancement of autophagy leads to increased viral glycoprotein synthesis → proviral	LC3 punctae formation	?	MRC-5 fibroblasts, MeWo cells	[46]
Autophagy in VZV infection is connected to the UPR and ER stress → most likely proviral	CREBH, ATF6β, PERK, BiP, DnaJB9/ERdj4, EDEM3, erasin, SEC62, ataxin-3, VCP/p97, INSIG, gp78, CHOP, HTRA4, C/EBPβ	Viral glycoproteins	MRC-5 fibroblasts, HeLa cells (transfected with viral glycoproteins)	[47]
Autophagic flux in VZV infection is not blocked → proviral	LC3 punctae formation	?	MRC-5 fibroblasts, human skin xenografts	[48]
Autophagy as mode of exocytosis for VZV → proviral	LC3, Rab11		MRC-5 fibroblasts	[49]
Late-stage autophagy is blocked in VZV infection, increased autophagy leads to lower viral titers, inhibition of autophagy leads to increased viral titers → antiviral	LC3 conversion		MRC-5 fibroblasts	[50]
Autophagy is upregulated upon VZV infection in a human organ culture model → most likely proviral	IL-6, LC3 punctae formation	?	Human skin organ culture	[51]
Autophagy as mode of exocytosis for VZV → proviral	LC3 conversion, LAMP1, LAMP2, Rab6, M6PR	?	MRC-5 and primary fibroblasts	[52]

## Data Availability

Not applicable.

## Abbreviations

ATG	Autophagyrelated protein
BECN	beclin
CNS	central nervous system
DEV	duck enteritis virus
ER	endoplasmic reticulum
FIP	focal adhesion kinase family interacting protein
HSV-1	Herpes Simplex Virus 1
M6PR	M6P receptor
MAP1-LC3	microtubule-associated protein light chain 3
mTOR	mechanistic target of rapamycin
3MA	3-methyladenine
ORF	open reading frame
PIK3C3	phosphatidylinositol 3-kinase catalytic subunit type 3 = VPS34
PRV	pseudorabies virus
SCID	severe combined immunodeficiency
ULK	unc-51-like-kinase
UPR	unfolded protein response
VZV	Varicella Zoster Virus
